# An Algorithm for Selecting Buoy Skin Paddle Design for Flap Monitoring in Total Autologous Breast Reconstruction After Nipple-Sparing Mastectomy

**DOI:** 10.7759/cureus.33443

**Published:** 2023-01-06

**Authors:** Stavros Samaras, Charles Malata

**Affiliations:** 1 Plastic Surgery, 401 Military Hospital of Athens, Athens, GRC; 2 Plastic and Reconstructive Surgery, Addenbrooke’s Hospital, Cambridge University Hospitals National Health Service (NHS) Foundation Trust, Cambridge, GBR; 3 Surgery, Addenbrooke’s Hospital, Cambridge University Hospitals National Health Service (NHS) Foundation Trust, Cambridge, GBR; 4 Surgery, Anglia Ruskin University School of Medicine, Chelmsford, GBR

**Keywords:** buried free flaps, skin-reducing mastectomy, nipple-sparing mastectomy, autologous breast reconstruction, free flap monitoring

## Abstract

Introduction

Monitoring buried flaps in reconstructive breast surgery is challenging, and the ideal technique is controversial. Established options include leaving an exterior (“buoy” or “sentinel”) skin paddle versus invasive implantable devices to avoid removing the paddle later. Technical modifications and an algorithm for strategic skin paddle positioning to circumvent this while avoiding complex monitoring equipment are proposed.

Patients and methods

Patients in whom buoy skin paddles were utilized for breast flap monitoring by a single surgeon were reviewed. Indications, demographic details, precise monitoring paddle location, and flap outcomes were evaluated. An algorithm and classification system were then formulated.

Results

Thirteen buoy skin paddles were utilized in seven patients (mean age: 43.5 years; range: 31-65) to monitor reconstructive flaps performed for risk-reducing mastectomies (four patients and seven breasts), therapeutic mastectomy (one breast), and revision surgery (three patients and five breasts). The flaps comprised seven deep inferior epigastric artery perforators (DIEPs), four superficial inferior epigastric arteries (SIEAs), and two pedicled latissimus dorsi (LDs) (mean free flap weight: 809 g; average mastectomy weight (n = 10 breasts): 467 g; range: 248-864). The skin paddles were located horizontally along the inframammary crease or vertically inferior to the nipple-areola or both. All flap transfers were successful with no re-explorations. All patients declined the monitoring paddle excision, and none have requested breast mound revision for poor cosmesis or contour deformities.

Conclusion

Vertical and horizontal skin paddles proved reliable for buried flap monitoring without recourse to invasive and expensive equipment. When designed appropriately, they do not require revision surgery. An algorithmic classification of skin paddle location to enable this is proposed.

## Introduction

Total autologous breast reconstruction following mastectomy predominantly utilizes free tissue transfers or pedicled flaps [1-3]. Conventional mastectomy techniques sacrifice a portion of the native breast skin (usually including the nipple-areola), which is then replaced by flap skin [4]. This cutaneous paddle allows effective monitoring of the flap [5,6]. In contrast, the more recently described nipple-sparing mastectomy (NSM) with its numerous advantages [7-11] preserves the entire breast envelope necessitating burying the whole reconstructive flap. Monitoring such buried breast flaps, especially free tissue transfers, is challenging, and the ideal technique is controversial. The success of free flaps, however, depends on the ability to quickly detect any anastomotic problems and swiftly return the patient to theater for flap exploration. Clinical examination complemented by handheld Doppler remains the gold standard for free flap monitoring, allowing some units to obtain flap salvage rates of up to 80%, with the overall success of flap transfer in the senior author’s series of more than 99% [12-14]. This is however dependent on having an external skin paddle to monitor both clinically and with a handheld Doppler.

In NSM or revision surgery with an adequate soft tissue envelope, the entire native skin is preserved, and therefore, a skin paddle is most often not necessary, making the monitoring of the buried flap a more challenging process. Over the years, numerous techniques and devices have been developed for free flap monitoring, including tissue oximetry, laser Doppler flowmetry, thermal imaging, external temperature measurements, implantable Doppler devices, and microdialysis [15-21]. However, none of these methods have been proven to be superior in terms of early detection of anastomotic problems compared to traditional monitoring of an exposed skin paddle with clinical examination and handheld Doppler [18-22]. Therefore, monitoring a flap with an exposed skin paddle remains the gold standard even after NSM autologous reconstruction. Detractors of leaving a monitoring skin paddle cite the need for a second operation to excise it postoperatively and prolonged hospital stay, the latter on the assumption that the excision takes place in the same admission [21]. Some authors routinely excise the so-called sentinel skin paddle one month after surgery [13]. Furthermore, attention has been drawn to the multiple revisions that are necessary for autologous reconstruction after NSM to improve aesthetic results [23,24].

Recent studies have therefore sought to evaluate revision surgeries following total autologous breast reconstruction after NSM to determine whether this was related to leaving a monitoring skin paddle or not. These have revealed comparable flap outcomes in totally buried free flaps versus those with external skin paddles for monitoring [25,26]. However, 60% of the patients with monitoring skin paddles needed them to be excised subsequently [26]. In addition, the literature is scant regarding the design and positioning of monitoring cutaneous paddles [13] with scarce written about how to select the skin paddle location (and incision) during NSM.

The present study examined outcomes of breast flap reconstruction in patients undergoing NSMs or revision reconstructions with adequate native skin envelopes. The technical modifications that circumvented the need for subsequent paddle removal by strategic positioning while avoiding expensive, complex, and potentially risky monitoring and allowing the gold standard method of clinical and handheld Doppler monitoring are presented. A classification and selection algorithm for skin paddle positioning during autologous reconstruction after nipple-sparing mastectomies is also proposed.

## Materials and methods

Study design

A chart review of all patients who underwent totally autologous breast reconstruction monitored with buoy skin paddles by a single surgeon (CM) from 2016 to 2020 was undertaken. Electronic medical records and photographs were evaluated to identify the indications, demographic details, precise monitoring paddle location, flap size, and overall outcomes. Secondary flap revisional operations searched for included return to theater for nipple-areola/mastectomy skin debridement, requirement for excision of the skin paddle or not, and liposuction or fat grafting to correct breast contour deformities. Donor site adjustments were excluded as they have no bearing on reconstruction after NSM. This project fell under the auspices of the Audit and Clinical Governance Department of Addenbrooke’s Hospital, Cambridge University Hospitals National Health Service (NHS) Foundation Trust, as an audit of the outcomes of free tissue transfers (2020) (registration number: PRN8444).

Patient selection

All planned mastectomies were discussed in the breast cancer (BRCA) oncoplastic multidisciplinary team (MDT) meeting with the participation of medical and radiation oncologists, radiologists, histopathologists, oncological breast surgeons, reconstructive plastic surgeons, and specialist nurse practitioners. NSM was offered to all females undergoing risk-reducing mastectomy (RRM) and to carefully selected patients with breast cancer. Patients were offered a choice between prosthetic and flap-based reconstruction. Incisions for the mastectomy and reconstruction including the location of monitoring skin paddles were carefully planned in conjunction with the breast surgeons both prior to and at the time of mastectomy.

Surgical technique

In all free tissue transfers, the internal mammary vessels were the recipient vessels of choice and were exposed to the rib-sparing technique. After the completion of the microsurgical anastomoses, the flaps were inset within the breast pocket and then deepithelialized leaving a well-planned skin paddle for monitoring, which would (ideally) include some of the cutaneous perforators. When the Doppler signal did not align with the desired skin paddle, minor adjustments to the flap inset or larger skin paddles were used to incorporate the signal. Drains, prophylactic antibiotics, and deep vein thrombosis (DVT) prophylaxis were used as per departmental protocol for free tissue transfer.

Flap monitoring

All flaps were monitored by trained nursing staff and residents using (the departmental policy that includes) an 8 MHz handheld Doppler and clinical observation hourly for the first postoperative day, two hourly for the next 48 hours, and four hourly thereafter until discharge. The clinical parameters recorded were flap color, temperature, capillary refill, flap consistency (turgor), and any bleeding or swelling as these can detect early warning signs before any change of the Doppler signal. No other adjunctive flap monitoring tests were used.

## Results

Over the four-year period, seven patients (mean age: 43.5 years; range: 31-65) had 13 “buoy” skin paddles to monitor their flap reconstructions (Table 1). The indications for surgery were risk-reducing mastectomies (four patients and seven breasts), therapeutic mastectomy (one breast), and revision surgery in three patients (five breasts). In terms of reconstructive timing with respect to mastectomy (six bilateral and one unilateral), four patients had immediate and the remaining three tertiary/salvage reconstruction for previous implant-based procedures. The latter comprised two patients post failed implant reconstructions: one following bilateral risk-reducing mastectomies and the other (20 years after quadrantectomy, adjuvant radiotherapy, and implant and thoracodorsal artery perforator {TDAP} flap revision) presented with intractable grade IV capsular contracture requiring capsulectomy and implant removal via an inframammary fold incision. The third patient had a history of bilateral skin-reducing mastectomies and implant-based reconstruction complicated by severe capsular contractures necessitating conversion to free tissue transfers. Even though the nipples were not preserved, she was included in this study to highlight the versatility of buoy skin paddles in breast reconstruction. The average mastectomy weight (n = 10 breasts) was 467 g (range: 248-864).

**Table 1:** Clinical summary of autologous breast reconstruction patients with buoy skin paddles DIEP, deep inferior epigastric artery perforator; IMF, inframammary fold; FTSG: full-thickness skin graft; SIEA, superficial inferior epigastric artery; NAC, nipple-areola complex; TDAP, thoracodorsal artery perforator; LD, latissimus dorsi; ADM, acellular dermal matrix; BRCA, breast cancer

Case	Age	BMI	Timing	Indication	Flap type	Incision	Skin paddle location	Breast weight (g)	Flap weights (g)	Revision surgery
1	35	26	Immediate	Risk reducing (strong family history)	Left: DIEP; right: DIEP	Left: IMF; right: IMF	Left: IMF; right: IMF	Left: 457 g; right: 405 g	Left: 683 g; right: 739 g	-
2	31	29.1	Immediate	Risk reducing (strong family history)	Left: DIEP; right: DIEP	Left and right: hemi-Y periareolar portion medial	Left: vertical; right: vertical	Left: 490 g; right: 529 g	Right: 879 g; left: 967 g	Patch of breast skin debrided + FTSG repair
3	31	30.6	Immediate	Risk reducing (BRCA mutation)	Left: SIEA; right: SIEA	Left and right: LeJour pattern (skin-reducing mastectomy)	Left: vertical (inferior to NAC); right: vertical (inferior to NAC)	Left: 864 g; right: 842 g	Right: 880 g; left: 895 g	Bilateral NAC necrosis - debrided + FTSG
4	52	25	Tertiary: post failed implants	Implant infection (history of risk-reducing mastectomy for BRCA mutation)	Left: DIEP; right: SIEA	Left and right: hemi-Y periareolar portion lateral	Left: vertical and periareolar; right: vertical and periareolar	Left: 355 g; right: 309 g	Right DIEP: 651 g; left SIEA: 533 g	-
5	65	29.6	Tertiary (status post TDAP and implant)	Grade IV capsular contracture	DIEP	IMF	IMF	-	Right DIEP: 726 g	-
6	35	22.5	Immediate	Left: cancer; right: risk reducing	Left: LD; right: LD	Left: IMF; right: IMF	Left: IMF; right: IMF	Right: 167 g; left: 248 g	-	-
7	55	29.1	Tertiary (status post implant-ADM reconstruction)	Bilateral severe (grades III and IV) capsular contracture	Left: SIEA; right: DIEP	Left: Wise pattern - previous T-junction excised including radiotherapy skin changes; right: vertical limb of previous T-junction	Right: vertical; left: horizontal	-	Right SIEA: 945 g; left DIEP: 1004 g	-
9	50		Tertiary (status post implant reconstruction)	Bilateral grade IV capsular contracture (history of risk-reducing mastectomy for BRCA mutation)	LD with implants	Bilateral LeJour pattern mammoplasty incisions	Right: vertical; left: vertical	Right: 449 g; left: 450 g	N/A	

**Figure 1:**
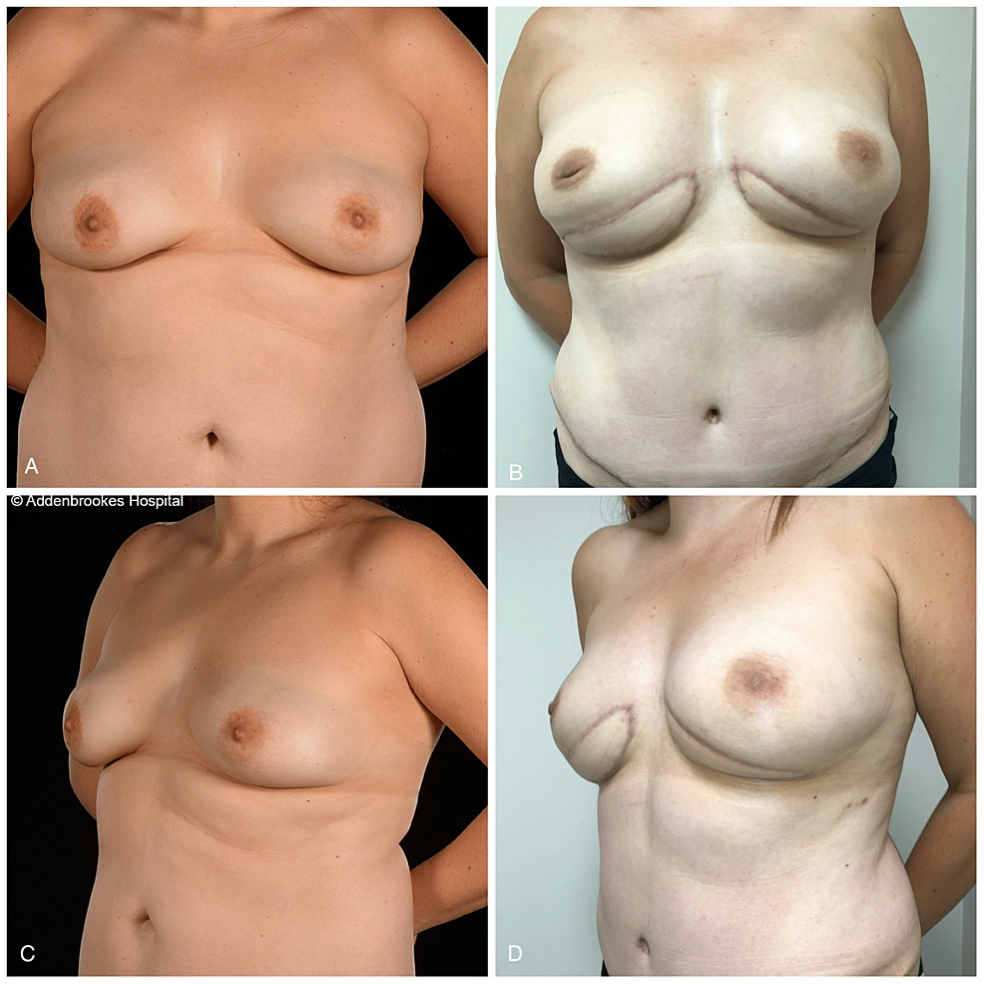
A 35-year-old with medial inframammary fold (IMF) skin paddles of free DIEP flaps A 35-year old with medial inframammary fold skin paddles of free DIEP flaps anastomosed to the internal mammary vessels following risk-reducing nipple-sparing mastectomies for strong family history (pre-op photos, A and C; appearances before and four months after surgery, B and D). As seen from the oblique photos, the IMF skin paddle was used to lengthen the short nipple-IMF distance enabling the creation of larger-sized breasts with the appropriate position of the nipples DIEP: deep inferior epigastric artery perforator

**Figure 2:**
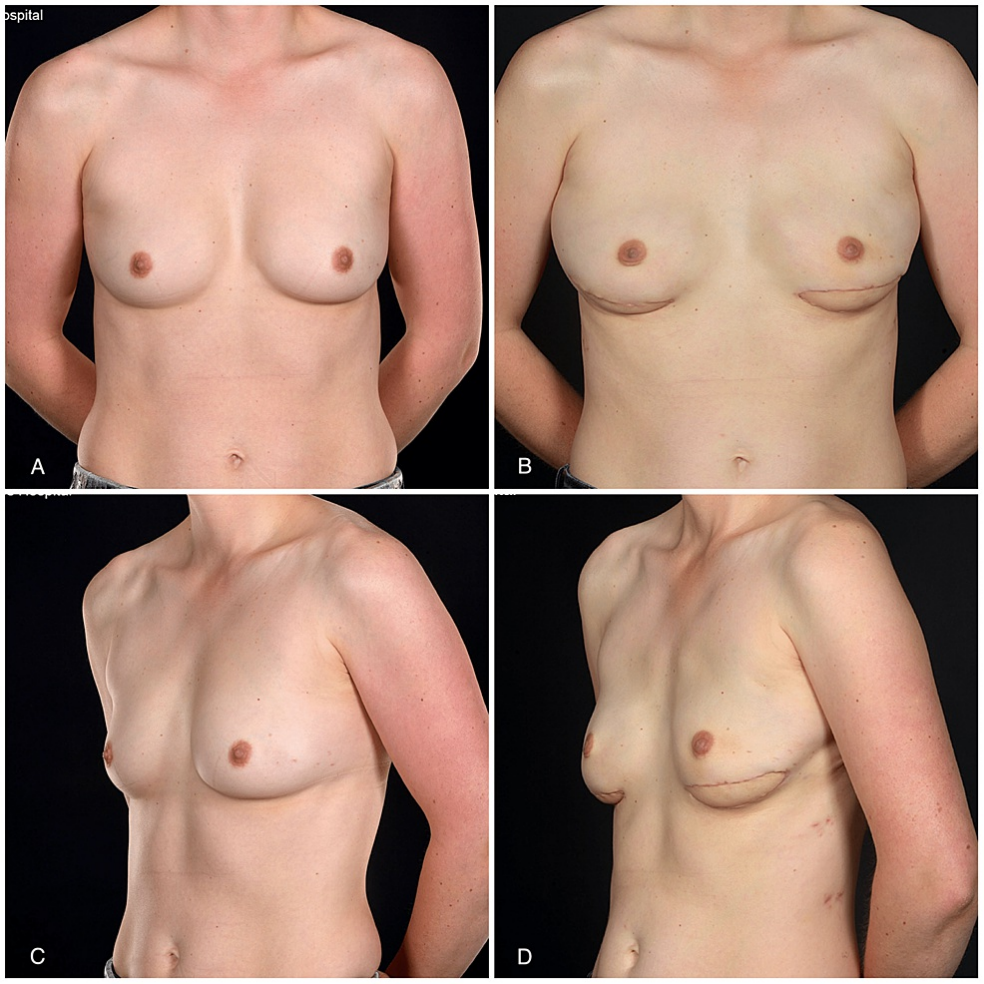
A 35-year-old with lateral inframammary fold (IMF) skin paddles of pedicled LD flaps This 35-year-old nulliparous patient with small nonptotic breasts (BMI: 22.5 kg/m^2^) underwent bilateral nipple-sparing mastectomy (left for cancer and right for risk reduction) and total autologous latissimus dorsi (LD) flap reconstruction with lateral IMF skin paddles (A and C, pre-op; B and D, post-op). The oblique views highlight the eccentric skin paddle locations to facilitate latissimus dorsi flap transposition. The photographs in the figure have been reproduced with kind permission from ePlasty (2018; 18:e13)

**Figure 3:**
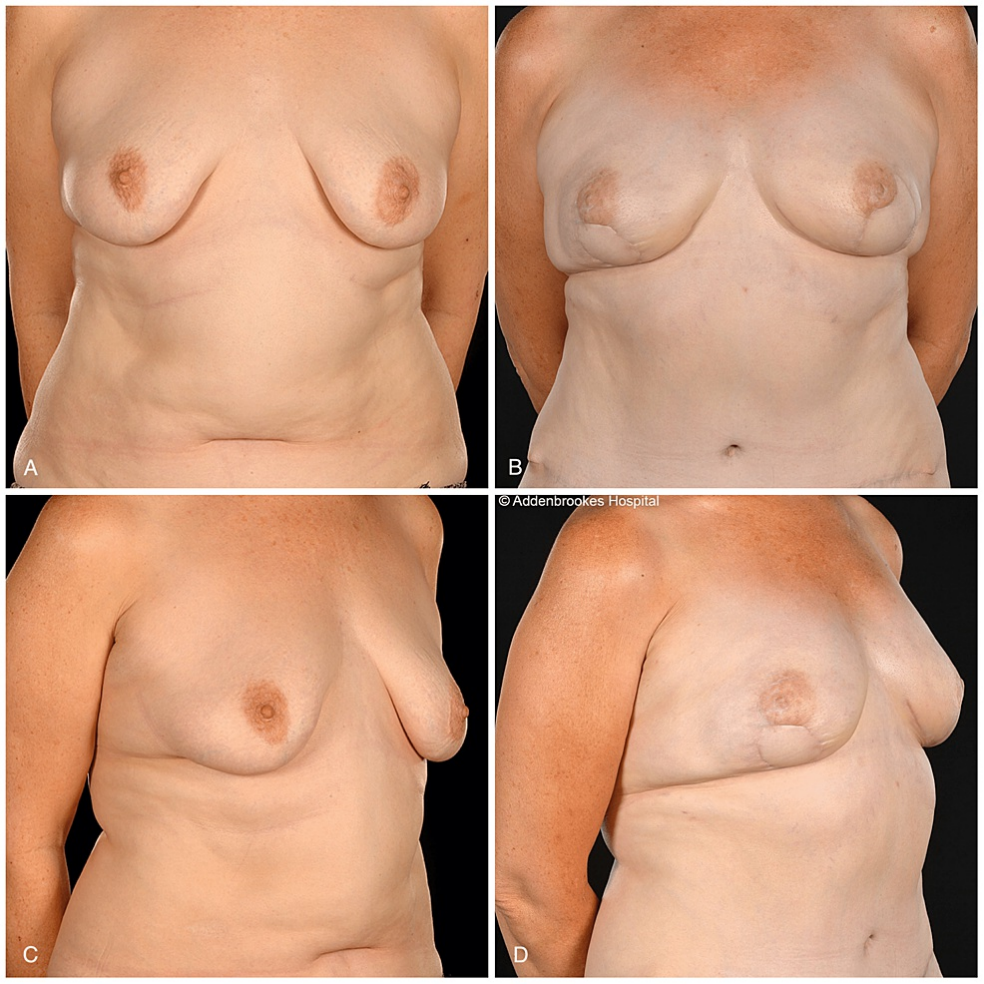
A 52-year-old with vertical skin paddles of free SIEA and DIEP flaps A 52-year-old patient (BMI: 25 kg/m^2^) with ptotic breasts underwent bilateral RRM and implant-ADM immediate breast reconstructions, which failed due to infection following a urinary tract infection. She subsequently underwent tertiary reconstruction with buried SIEA and DIEP flaps (pre-op views: A and C). Vertical buoy skin paddles were used with lateral periareolar extensions. Despite the improved breast shape (versus her preoperative status), she requested fat grafting for volume insufficiency and the correction of left breast lateral indentation. She declined the excision of the skin paddles at the same time (post-op views: B and D) SIEA, superficial inferior epigastric artery; DIEP, deep inferior epigastric artery perforator; RRM, risk-reducing mastectomy; ADM, acellular dermal matrix

**Figure 4:**
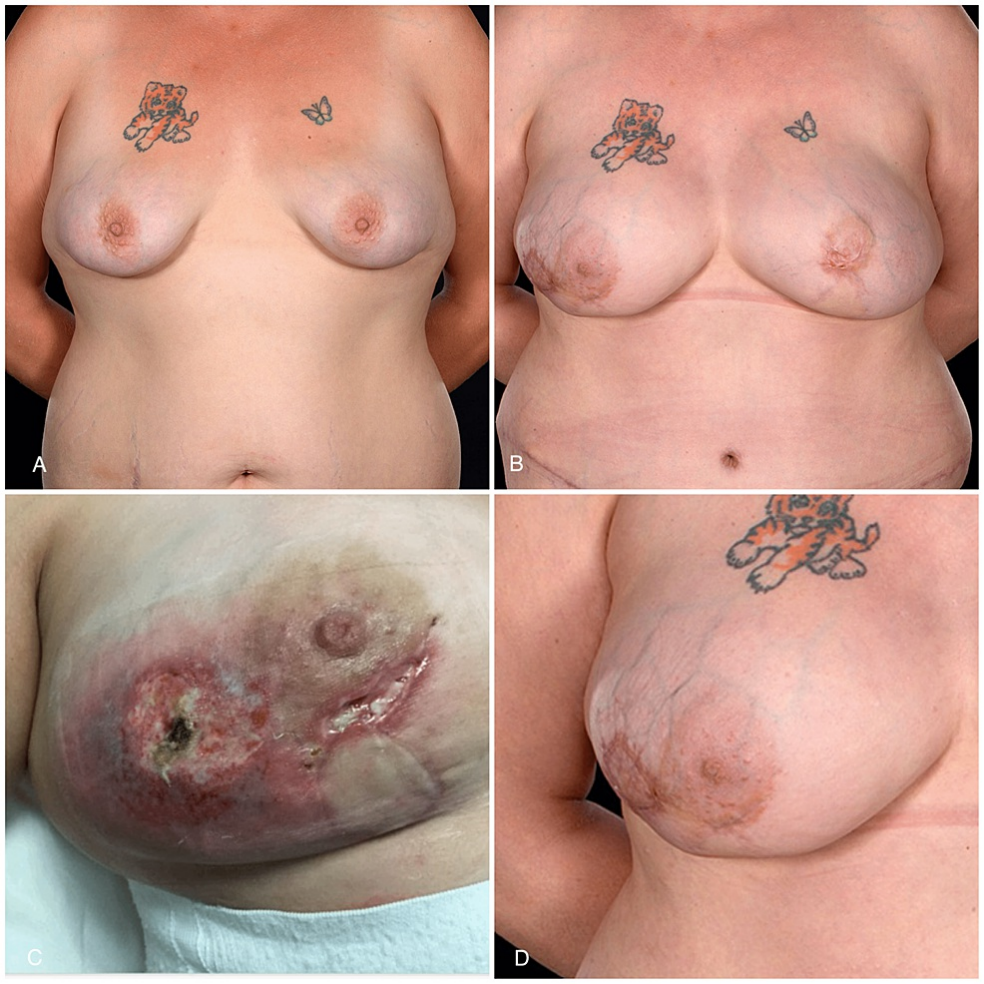
A 31-year-old with vertical skin paddles of free DIEP flaps A 31-year-old smoker (BMI: 29.1 kg/m^2^) underwent bilateral RRM and reconstruction with DIEP flaps (pre-op view: A). She developed a patch of mastectomy skin flap necrosis laterally on the right side and partial nipple necrosis on the left (C). The mastectomy patch was treated with a full-thickness skin graft (harvested from the abdominal dog ear) eight weeks after reconstruction and healed well (post-op views: B and D) with the preservation of the contour of her breast mound. Note that the buoy skin paddles were used to widen and enlarge the breasts, correct their tuberous component, and narrow the wide intermammary distance DIEP, deep inferior epigastric artery perforator; RRM, risk-reducing mastectomy

The reconstructions comprised seven deep inferior epigastric artery perforators (DIEPs), four superficial inferior epigastric arteries (SIEAs), and two pedicled latissimus dorsi (LD) musculocutaneous flaps (mean free flap weight: 809 g). The mean BMI in patients undergoing free flap reconstruction was 28.2 kg/m^2^ (range: 25-30.6), whereas the LD flap patient’s BMI was only 22.5 kg/m^2^. The skin paddles were located “horizontally” (in various positions) along the inframammary crease or vertically inferior to the nipple-areola or both (Table 1; Figures 1-4). All flap transfers were successful with no re-explorations. Postoperatively, all patients declined the excision of the monitoring skin paddles, and none have requested breast mound revision for poor cosmesis or contour deformities.

The indications for the risk-reducing mastectomies were strong family history in two patients, BRCA mutation in two patients (one with tertiary reconstruction), and contralateral breast cancer in one patient. For four of the mastectomies (two patients), inframammary (fold) incisions were used (Figure 1), and the remaining four mastectomies (two patients) were accomplished via hemi-Y periareolar incisions, with the periareolar portion being medial in two breasts and lateral in the remaining two. Furthermore, one patient has had bilateral LeJour pattern skin-reducing nipple-sparing mastectomy with vertical monitoring skin paddles inferior to the areolae.

The skin paddle positioning followed the mastectomy incision pattern. In six flaps (four patients), inframammary skin paddles were used (Figure 2), whereas for the remaining seven flaps, a vertical buoy skin paddle inferior to the nipple-areola complex (NAC) was utilized (Figure 3). In one patient (two breasts) with hemi-Y periareolar incisions, the vertical skin paddle was used not only for monitoring purposes but also to correct the tuberous components of the breasts (Figure 4). In the patient who had explantation of the prostheses and tertiary reconstruction with contralateral DIEP and SIEA flaps, the buoy skin paddles were positioned vertically and horizontally, respectively. In this patient, the vertical skin paddle was used to widen the breast as she was status post breast reduction, and on the left breast, an inferior melon slice of skin was excised to incorporate the old T-scar along with scarring from previous radiotherapy. The latter was replaced with a horizontal buoy skin paddle.

Revisional procedures were required in four patients postoperatively. One free flap patient (BMI: 31) with macromastia (mastectomy and flap weights as charted in Table 1) developed bilateral nipple-areola complex (NAC) necrosis, which required debridement and reconstruction of the defects with full-thickness skin grafts (FTSG) harvested from her abdominal dog ears. One heavy smoker experienced partial nipple necrosis, which was successfully managed conservatively (dressings) with excellent cosmetic outcome (Figure 4). She also had full-thickness necrosis of the part of the contralateral mastectomy skin flap, which was debrided and reconstructed with FTSG (Figure 4C). One of the patients has subsequently undergone single-stage fat grafting to both breasts for volume insufficiency (Figure 3).

There were no partial or total flap losses, and all the skin paddles survived. There were no flap re-explorations. Notably, none of the patients requested/agreed to excision (or deepithelialization and burying) of the buoy skin paddle even though this was offered to all of them prior to and after the reconstruction. The patient with the NSM for breast cancer (immediate reconstruction with pedicled LD flaps) developed metastatic disease, which was ultimately fatal but without any local recurrence identified (left multifocal grade 3 invasive cancer no special type {NST}, N0 {0/4 sentinel lymph node biopsy}, estrogen receptor {ER} positive 8/8, and human epidermal growth factor receptor-2 {HER2} negative). 

## Discussion

In standard mastectomy techniques, a skin paddle is designed to replace the resected central mastectomy skin incorporating the nipple-areola. It thus improves the aesthetic appearance of the reconstructed breast and at the same time allows flap monitoring with clinical examination and handheld Doppler probes. However, in nipple-sparing mastectomy (NSM), the nipple-areola complex is spared, and as such, there is no need for an external skin paddle, which can be used for monitoring. Despite the evolution of various flap-monitoring techniques, none of these have proved superior to the standard clinical examination in conjunction with handheld Doppler [15-22]. This gold standard method however requires the presence of an accessible and visible patch of flap skin to use for monitoring. This makes monitoring completely buried flaps particularly challenging. This problem was first recognized in head and neck reconstruction where completely buried flaps are common and the effects of a potential flap failure more detrimental. Consequently, the idea of externalizing a skin paddle for the sole purpose of flap monitoring, which could then be excised at a second stage, was developed for jejunal and fasciocutaneous flaps used in pharyngeal and laryngeal reconstruction [27-29].

This technique was adopted for autologous tissue breast reconstruction monitoring after our oncological breast surgeons started performing NSM and we elected to leave a “buoy” or “sentinel” skin paddle for flap monitoring. In this limited series of seven patients, 13 flaps were performed for immediate NSM and revisional reconstructions with monitoring provided by variously located external skin paddles. The results suggest that if the skin flap is carefully designed, it can blend in with the native breast contours/shadows and circumvent the need for excision or revision surgery to the breast mounds on the account of contour deficiencies. This principle was successfully applied to both free tissue and pedicled flap reconstructions. The different skin paddle color from the native breast skin apparently did not adversely affect the aesthetics of the reconstruction as perceived by the patients.

**Figure 5:**
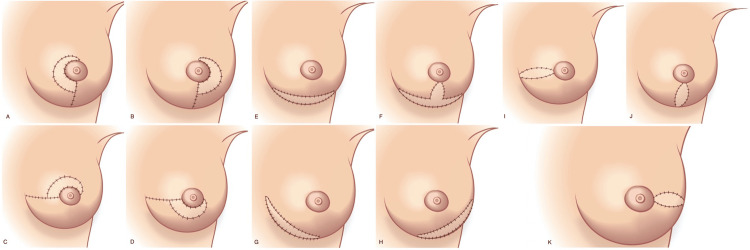
Diagrammatic illustration of skin paddle locations in autologous breast reconstruction following nipple-sparing mastectomy. The key features of each design are shown (A) Medial periareolar paddle (± inferior extension): facilitates access to internal mammary vessels. (B) Lateral periareolar paddle (± inferior extension): for access to the axilla. (C) Superior periareolar paddle location (± medial or lateral extension): suffers from visibility problems and like all periareolar incisions risk of devascularization of the nipple-areola complex; might be useful if the distance between the nipple and superior border of the breast is short, e.g., previous superior lumpectomy scar with skin deficit or retraction. (D) Inferior periareolar paddle location (± medial or lateral extension): suffers from visibility problems and like all periareolar incisions risk of devascularization of the nipple-areola complex; might be useful if the distance between the nipple and IMF is short, e.g., previous inferior lumpectomy scar with skin deficit or retraction. (E) Central inframammary paddle location: versatile especially in small breasts with short N-IMF distances. (F) Wise or inverted-T pattern/combined vertico-inframammary paddle position: very versatile, used to widen the breast, can lengthen N-IMF distance, ideal in previous breast reductions, takes tension off the T-junction, very useful for Wise pattern skin-reducing mastectomies. (G) Medial-central IMF paddle location: for microsurgical access to IMVs. (H) Lateral-central IMF paddle location: facilitates microsurgical access to thoracodorsal vessels and pedicled latissimus dorsi flap transposition. (I) Radial lateral (horizontal) paddle: for microsurgical access to TDVs, can also be extended around ¼ or ½ of the areola inferiorly or superiorly. (J) Radial inferior (vertical) skin paddle: widens the breast and heals very well, avoids a T-junction, very versatile, can be extended to the IMF or partially around ¼ to ½ of the areolar diameter (to create a hemi-Y incision). (K) Radial medial (horizontal) paddle: not very practical as can be obvious and thus necessitate excision. Credits: SS

From the results of the present study, a proposed nomenclature system for classifying the skin paddle location is illustrated in Figure 5. The skin paddle positioning followed the (mastectomy) incision pattern. In six flaps, the skin paddle was hidden within the inframammary fold, whereas in the rest, it was positioned vertically below or adjacent to the NAC. In one of these patients, the vertical skin paddle was carefully planned to elongate and widen the inferior pole of the breast and thus correct the tuberous deformity (Figure 4). None of the patients requested or agreed to have their monitoring skin paddles removed as they are unobtrusive enough.

The greatest benefit of careful positioning of the skin paddle is the possibility of an immediate autologous tissue single-stage breast reconstruction following NSM, which circumvents the need for subsequent paddle removal. Non-flap-threatening complications were observed in three of the 13 reconstructed breasts (bilateral nipple necrosis in one patient and a patch of mastectomy skin flap necrosis in another). Both were treated by full-thickness skin grafting. Notably, none of the patients herein reported requested the removal of the skin paddles that had been used for monitoring at the time of the revisional procedures or at any other time. Our findings of the need for strategic positioning of the buoy skin paddle to avoid the requirement to remove it are further supported by a recent study that compared revision rates in completely buried free flaps (monitored by implantable Doppler) and free flaps with skin paddle in patients undergoing NSM, which showed that almost 60% of the group with skin paddles requested excision of the monitoring skin [26].

In our technical considerations, we would advise ideally positioning the skin paddles away from the nipples to avoid the distortion of the NAC proper and its inadvertent devascularization. It is also important to undertake judicious care of the nipple-areola during the mastectomy and reconstruction prior to wound closure. We experienced bilateral nipple necrosis in an obese patient with periareolar deepithelialization carried out at the beginning of the surgery as part of the LeJour pattern nipple-sparing mastectomy, and we surmised that the disruption of the subepidermal vascular plexuses in combination with the mastectomy has generated the necrosis. The dermis has become desiccated during the long bilateral free flap procedure, and so now, we avoid deepithelialization at the time of the mastectomy leaving it to be done at the flap inset. Our experience is consistent with the recent NSM literature where the periareolar and Wise pattern incisions have been correlated with higher rates of NAC necrosis compared to IMF incisions [1,30]. As this patient had a BMI of 30.6 kg/m^2^ with macromastia, maybe a staged approach to the nipple preservation with a prior mastopexy, for instance, would be a safer strategy.

“Horizontally” orientated skin paddles along the inframammary crease can be located eccentrically in order to improve access for the microsurgical anastomoses: more medially for the internal mammary vessels or more laterally for the thoracodorsal vessels. The latter should also be used for LD flap reconstructions to facilitate flap transposition. The “horizontal” skin paddle can be hidden in the natural silhouette of the inferior pole of the breast, and at the same time, it can be used to lengthen a short nipple-IMF distance (Figure 2).

**Figure 6:**
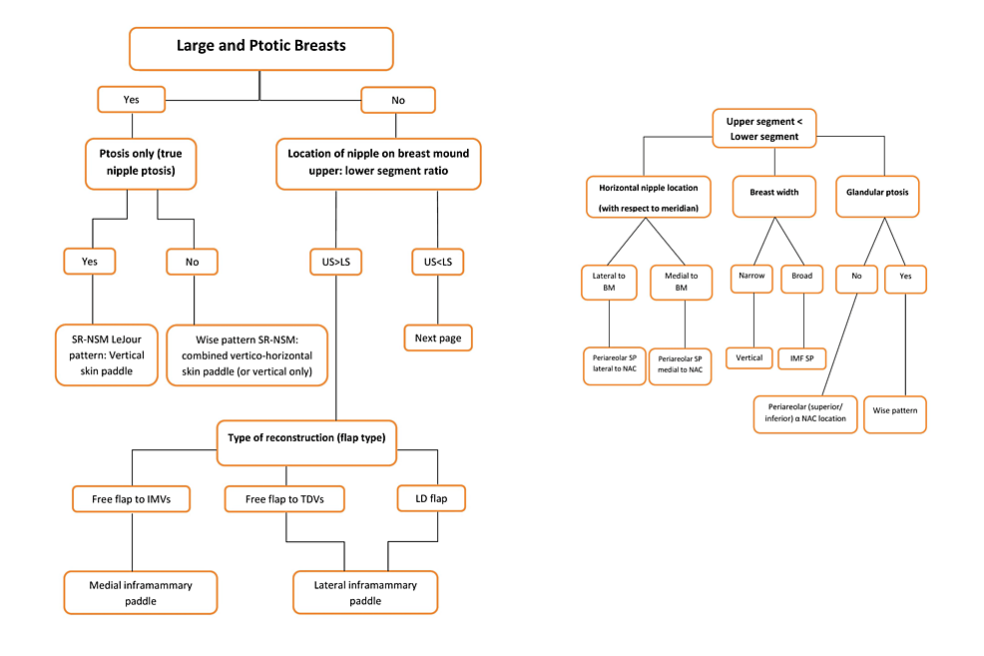
An algorithmic illustration of skin paddle design in autologous breast reconstruction following nipple-sparing mastectomy SR-NSM, skin-reducing nipple-sparing mastectomy; US, upper segment; LS, lower segment; IMVs, internal mammary vessels; TDVs, thoracodorsal vessels; LD, latissimus dorsi; BM, breast meridian; SP, skin paddle; NAC, nipple-areola complex; IMF, inframammary fold; α, dependent on

Vertical (radial) infra-areolar skin paddles are versatile as they can be used in various nipple-sparing mastectomy incisions such as hemi-Y and LeJour pattern or in tertiary reconstructions with a history of skin reduction (previous breast reduction or mastopexy) in order to widen the breast. In addition, it can replace a radial lumpectomy scar from previous radiation [13]. Moreover, a combined vertico-IMF buoy skin paddle (anchor-shaped or Wise pattern type) can be used to replace previous inverted-T incisions with the benefit of increasing both the width and the height of the breast. Care must be exercised in skin-reducing mastectomies so as not to devascularize the NAC or allow it to desiccate especially under the microscope and to avoid any tension on the skin flaps including the NAC. Another practical location for a skin paddle is laterally after a radial incision for the mastectomy. This is particularly useful if axillary surgery is also being undertaken. Radial incisions can be extended around the areola to improve access for the mastectomy and the flap reconstruction. However, care must be taken not to extend this injudiciously as it might impair nipple-areola viability. An algorithm for selecting skin paddle location and hence mastectomy incision is given in Figure 6. The key factors that determine these choices are summarized in Table 2.

**Table 2:** Factors in decision-making: nipple-sparing mastectomy incision and monitoring paddle location Credits: CM

Factors in decision-making	
Size of the breast to be reconstructed	Small breast: inframammary fold skin paddle is preferable
Large, ptotic breast	Vertical pattern skin paddle, Wise pattern, or combined vertico-horizontal (T or anchor shaped) paddle
Location of the nipple on breast mound	“Short” lower segment: (horizontal) inframammary fold skin paddle; “long” lower segment: Wise pattern skin paddle and vertical pattern skin paddle
Breast width	Narrow: vertically oriented skin paddle preferable
Location of the nipple with respect to breast meridian	Lateral: periareolar skin paddle lateral to the nipple-areola complex; medial: periareolar skin paddle medial to the nipple-areola complex

The current study has certain limitations, including its retrospective design, with data derived from a single surgeon’s practice (senior author: CM) with a small index sample size. A multi-institutional (national) audit would address some of these limitations where a larger sample group could give us the opportunity to compare the revision rates of buoy skin paddles and standard skin paddles in breast reconstruction. Furthermore, when we conducted our study, the follow-up was limited to 24 months postoperatively. However, as mentioned earlier, none of the patients requested the removal of the skin paddles at the time of the revisional procedures or any other time.

## Conclusions

In conclusion, this small retrospective series of a single oncological and reconstructive surgical team demonstrated that with careful skin paddle position, monitoring buoy flaps need not be subsequently resected as they were well accepted by patients. The algorithm presented can assist plastic surgeons in the decision-making process of selecting the design and location of the sentinel skin paddle, thus enabling standard monitoring without recourse to invasive and expensive equipment.
